# Can Radiofrequency Ablation Replace Liver Resection for Solitary Colorectal Liver Metastasis? A Systemic Review and Meta-Analysis

**DOI:** 10.3389/fonc.2020.561669

**Published:** 2020-11-17

**Authors:** Wu Hao, Jiang Binbin, Yang Wei, Yan Kun

**Affiliations:** Key Laboratory of Carcinogenesis and Translational Research (Ministry of Education/Beijing), Department of Ultrasound, Peking University Cancer Hospital and Institute, Beijing, China

**Keywords:** liver resection, radiofrequency ablation, colorectal liver metastasis, therapeutic efficacy, meta-analysis

## Abstract

Radiofrequency ablation (RFA) can be a favorable option for patients with colorectal liver metastasis (CRLM). However, current reports about the therapeutic efficacy of liver resection (LR) and RFA for colorectal liver metastasis (CRLM) still remain controversial, especially for solitary CRLM. Therefore, this meta-analysis was performed to evaluate the therapeutic efficacy between LR and RFA for solitary CRLM. First, a comprehensive search for published studies was conducted using PubMed, the Cochrane Library Central, and Web of Science. Each study was reviewed and data extracted. In this meta-analysis, 10 studies (11 study arms) were finally included. The meta-analysis was performed using risk ratio (RR) and random effect model or fixed effect model, in which 95% confidence intervals (95% CI) for RR were calculated. The primary outcomes were disease-free survival (DFS) and overall survival (OS) at 1, 3, or 5 years plus complication rate. The results showed that patients treated by LR achieved better PFS and OS than those by RFA, but subgroup analysis and meta-regression displayed that the efficacy of RFA was equivalent to that of LR in solitary CRLM, when conditions were limited to tumors of ≤ 3 cm and fewer synchronous metastasis in the publication years 2011–2018. Meanwhile, RFA achieved lower complication rates when compared with LR. In conclusion, although patients treated by RFA cannot achieve better PFS and OS than those by LR, RFA can be considered a viable treatment option for solitary CRLM, with potentially lower complication rates.

## Introduction

Colorectal cancer has become one of the most common human malignancies, affecting nearly 1 million individuals in the world every year (1, 2). When the colorectal cancer was diagnosed, up to half of the patients developed colorectal liver metastases (CRLM) (3). Colorectal liver metastasis significantly affects overall survival (OS), which has become the leading cause of cancer-related mortality in patients with colorectal cancer; the median OS for patients with untreated CRLM is 4.5–12 months (4, 5). Currently, liver resection (LR) is considered as the most effective treatment approach for CRLMs. However, only 10–30% of the cases are considered eligible for surgical resection because of general health status, anatomical location, disease extent, hepatic function reservation, or comorbidities (6–8).

Radiofrequency ablation (RFA), as a common minimally invasive treatment modality, has been widely used in clinical practice for the local control of liver tumors, and previous reports have demonstrated that thermal ablation had an advantage over surgical resection in being less invasive for hepatocellular carcinoma (HCC) (≤ 3 cm); therefore, it can also be an alternative option for patients with unresectable CRLM (9–14). Although RFA has established its role in the management of HCC as a safe, well-tolerated, and less invasive procedure, there has been no consensus on the therapeutic efficacy of RFA for those patients with CRLM, especially for solitary lesions (15–18).

In recent years, several studies about solitary CRLM reported a comparable OS and complication rates for RFA vs. LR. These results have led to the discussion that RFA should be favored over LR due to its less invasive and easily repeated procedure, yet RFA for patients with unresectable CRLM has been labeled inferior to LR for patients with resectable CRLM according to previous studies (17, 19). However, these results should be interpreted with caution because of the apparent selection bias.

Along this line, this study analyzed the existing literature comparing the therapeutic efficacy and safety of RFA and LR for patients with solitary CRLM by conducting a meta-analysis and analyzed the factors influencing prognosis to evaluate noninferiority or inferiority of RFA for patients with unresectable CRLM.

## Methods

### Literature Search

The QUOROM guidelines were followed for conducting the meta-analysis. The systematic literature search was performed independently by two of the authors using PubMed, Web of Science, and the Cochrane Library Central. No restriction was set for the date of publication. Only studies on humans and in the English language were considered for inclusion. The following Medical Subject Heading terms (MeSH) search headings were used: “radiofrequency ablation,” “resection,” “surgical treatment,” “surgery,” “hepatectomy,” “colorectal tumor,” “colorectal neoplasm,” “colorectal cancer,” “liver,” “hepatic,” “metastases,” and “metastasis.” The computer program Endnote X7 was used for reference management.

### Inclusion Criteria

For inclusion in the meta-analysis, the study had to fulfill the following criteria: (1) the comparative studies of clinical outcomes between RFA and LR for solitary CRLM; (2) the studies reporting at least 1-year disease-free survival and 3- or 5-year overall survival of each treatment group; (3) the studies clearly document indications for RFA and HR; (4) when more than one study were reported by the same research, the one of higher quality or the most recent publication was included.

### Exclusion Criteria

The following studies (cohorts) were excluded: (1) the original studies lacking the comparative results about the clinical outcomes of RFA and LR; (2) those studies published in the form of review articles (including meta-analysis), abstracts, comments, letters, editorials, and case reports.

### Data Extraction

Data extraction was performed independently by Wu Hao and Jiang Binbin, and in the case of discrepancy, the decision was made by a discussion with a third author (Yan Kun). For literatures with no clear survival data, data extraction was performed in the survival curve from primary literature by the Engauge digitizer software. The following parameters from each study were extracted: (1) the first author, the year of publication, study design; (2) the baseline oncological characteristics of patients including tumor size, tumor count, study period, primary lymph nodes, adjuvant chemotherapy, timing of metastasis; and (3) the outcome of the trials including 1-year disease-free survival (DFS), 3- and 5-year overall survival (OS), and complications.

### Statistical Analysis

All analyses were performed using the STATA statistical software package version 12.0 (STATA Corp., College Station, Texas, USA). Calculation for dichotomous variables was carried out using the estimation of risk ratio (RR) with a 95% confidence interval (95% CI). The pooled effect was calculated using either the fixed effects model or the random effects model. The heterogeneity among the included studies was evaluated by the I2 statistics and Chi-squared test. In addition, the heterogeneity was considered to be present if the I2 was more than 50%. Sensitivity analysis was performed to evaluate the stability of the results by subgroup analysis and meta-regression analysis. Evidence of publication bias was evaluated using the Begg's test. Two-sided *P* < 0.05 was considered statistically significant.

## Results

### Selection of Trials

**Figure 1:**
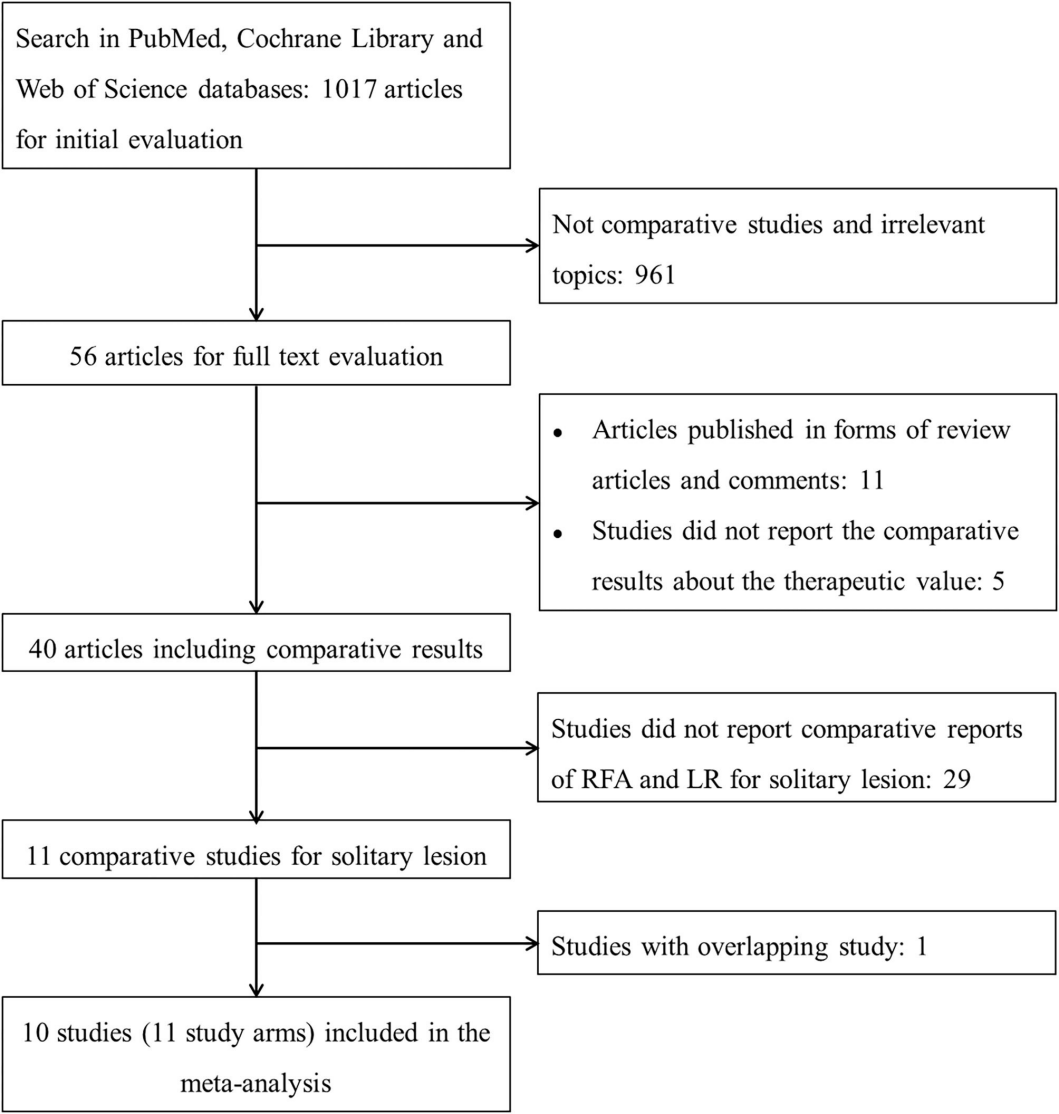
The flowchart describing the selection and exclusion of the existing literature. RFA, radiofrequency ablation; LR, liver resection.

After initial screening, 11 potentially relevant clinical study cohorts were identified. Of them, two study cohorts were from the same medical center, the latest one with the most comprehensive information was enrolled. Thus, a total of 10 study cohorts (11 study arms) with sample size ranging from 29 to 226 have been enrolled (Figure 1) (2, 15–18, 20–24). Among them, 690 patients underwent LR, and 347 patients underwent RFA. A detailed information of the included studies is summarized in Table 1.

**Table 1:** Characteristics of the included studies.

**References**	**Publication**	**Geographic**	**Tumor size**	**RFA method**	**AC**	**SM**	**Complications**	**Sample size**	**Reason of**
	**year**	**location**	**(RFA/LR, cm)**		**(RFA/LR)**	**(RFA/LR)**	**(RFA/LR)**	**(RFA/LR)**	**(RFA/LR)**
Oshowo et al. (15)	2003	European	3/4	perc	23/17	14/4	1/2	25/20	1,2,4
Aloia et al. (25)	2006	American	3/3.5	intra	24/99	18/74	NA/NA	30/150	2,3
Berber et al. (26)	2008	American	3.7/3.8	intra	30/63	5/15	2/28	42/90	2,4
Hur et al. (17)	2009	Asian	2.5/2.8	NA	22/37	7/24	0/6	25/42	1,2,3
Kim et al. (21)	2011	Asian	1.7/1.4	NA	90/114	9/104	4/26	99/127	5,6,7
Kim et al. (21)	2011	Asian	3.6/4.8	NA	14/50	1/34	1/3	14/56	5,6,7
Ko et al. (22)	2014	Asian	2.02/3.59	perc	8/6	5/3	NA/NA	17/12	4,6
Lee et al. (27)	2015	Asian	1.8/1.7	intra	26/52	19/47	5/28	29/63	2,3
McKay et al. (24)	2009	American	3.0/4.1	intra	NA/NA	NA/NA	8/22	19/37	2,8
Takahashi et al. (28)	2018	American	1.81/1.91	intra	NA/NA	NA/NA	NA/NA	25/63	3
White et al. (29)	2007	American	2.4/2.7	perc	11/20	5/17	1/4	22/30	9

RFA, radiofrequency ablation; LR, liver resection; AC, adjuvant chemotherapy; SM, synchronous metastasis; Perc, percutaneous RFA; Intra, intraoperative RFA.

*Reason of RFA: 1, close to major vessel; 2, prohibitive comorbidity; 3, inadequate liver remnant; 4, extrahepatic disease; 5, difficult anatomical location; 6, multiple metastasis; 7, severe cardiovascular or pulmonary disease; 8, proximity to critical structures; 9,other reasons with no eligible for liver resection*.

### Efficacy

**Figure 2:**
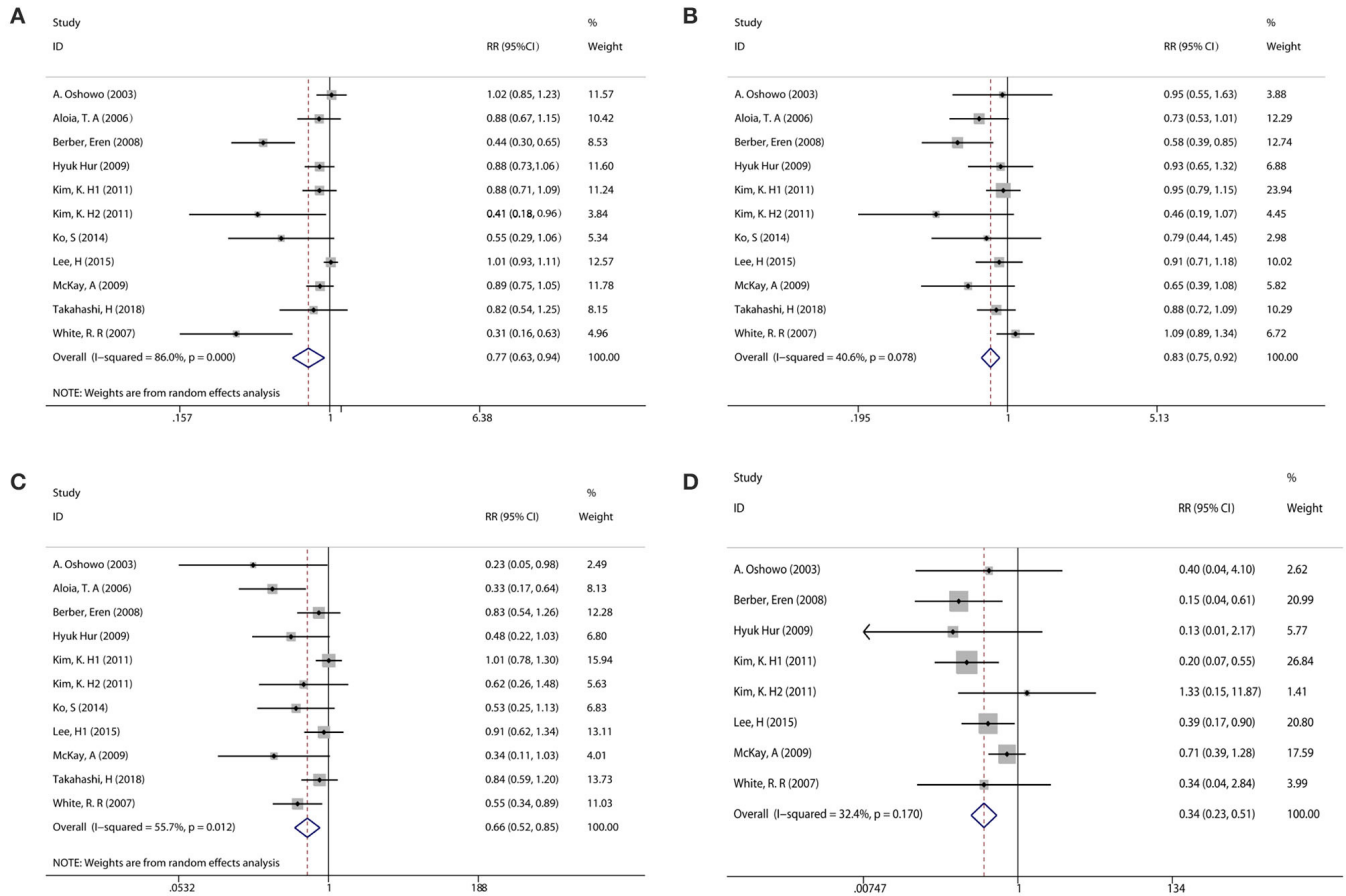
Pooled analysis comparing the survival rate between RFA and LR groups. **(A)** Pooled analysis comparing the 1-year PFS rate. **(B)** Pooled analysis comparing the 3-year overall survival (OS) rate. **(C)** Pooled analysis comparing the 5-year OS rate. **(D)** Pooled analysis comparing the complication rate.

With observable interstudy heterogeneity, patients in the RFA group had slightly inferior 1-year PFS (RR: 0.77, 95% CI: 0.630–0.940, *P* = 0.009, *I*^2^ = 86.0%, Ph = 0.000) (Figure 2A, Table 2), 3-year OS (RR: 0.860, 95% CI: 0.760–0.980, *P* = 0.021, *I*^2^ = 40.6%, Ph = 0.078) (Figure 2B, Table 2), and 5-year OS (RR: 0.66, 95% CI:0.52–0.85, *P* = 0.001, *I*^2^ = 55.7%, Ph = 0.012) (Figure 2C, Table 2) when compared with patients in the LR group.

**Table 2:** Subgroup analysis and meta-regression.

**Analysis**	***n***	***Weight***	***HR (95%CI)***	***P***	***I2***	***Ph***	***Pr***
**1-y PFS**	11	100%	0.77 (0.63–0.94)	0.009	86.0%	0.000	
Publication year							0.823
Year (2003–2010)	6	41.87%	0.86 (0.79–0.95)	0.002	77.9%	0.000	
Year (2011–2018)	5	58.13%	0.97 (0.90–1.05)	0.453	56.8%	0.055	
Geographic location							0.902
Asian	5	66.80%	0.96 (0.89–1.03)	0.283	58.3%	0.048	
American	5	22.76%	0.79 (0.70–0.90)	0.000	77.3%	0.001	
European	1	10.45%	1.02 (0.85–1.23)	0.817	–	–	
Tumor size for RFA							0.014
≤ 3 cm	9	97.16%	0.95 (0.89–1.00)	0.071	54.5%	0.025	
>3 cm	2	2.84%	0.43 (0.30–0.62)	0.000	0.0%	0.885	
RFA methods							0.517^*^
Percutaneous	3	14.93%	0.91 (0.76–1.08)	0.285	84.3%	0.002	
Intraoperative	5	85.07%	0.95 (0.88–1.02)	0.129	78.0%	0.001	
Adjuvant chemotherapy							0.406^*^
PAC_RFA_ > PAC_LR_	6	85.58%	0.96 (0.89–1.03)	0.224	77.2%	0.001	
PAC_RFA_ ≤ PAC_LR_	3	14.42%	0.80 (0.68–0.95)	0.012	78.4%	0.010	
Synchronous metastasis							0.485^*^
PSM_RFA_ > PSM_LR_	3	19.12%	0.94 (0.81–1.10)	0.451	44.7%	0.164	
PSM_RFA_ ≤ PSM_LR_	6	80.88%	0.93 (0.87–1.00)	0.051	83.9%	0.000	
**3-y OS**	11	100%	0.86 (0.76–0.98)	0.021	40.6%	0.078	
Publication year							0.710
Year (2003–2010)	6	43.8%	0.88 (0.77–1.01)	0.065	55.6%	0.046	
Year (2011–2018)	5	56.2%	0.90 (0.80–1.02)	0.093	0.0%	0.558	
Geographic location							0.781
Asian	5	44.52%	0.91 (0.80–1.05)	0.187	0.0%	0.565	
American	5	52.78%	0.87 (0.77–0.99)	0.031	64.0%	0.025	
European	1	2.7%	0.95 (0.55–1.63)	0.841	–	–	
Tumor size for RFA							**0.024**
≤ 3 cm	9	93.6%	0.92 (0.84–1.01)	0.086	0.0%	0.561	
>3 cm	2	6.4%	0.56 (0.39–0.79)	0.001	0.0%	0.623	
RFA methods							0.101^*^
Percutaneous	3	33.89%	1.04 (0.87–1.25)	0.666	0.0%	0.579	
Intraoperative	5	66.11%	0.81 (0.71–0.92)	0.001	26.1%	0.247	
Adjuvant chemotherapy							0.210^*^
PAC_RFA_ > PAC_LR_	6	65.38%	0.85 (0.75–0.96)	0.10	40.9%	0.133	
PAC_RFA_ ≤ PAC_LR_	3	34.62%	1.02 (0.86–1.22)	0.791	0.0%	0.511	
Synchronous metastasis							0.516^*^
PSM_RFA_ > PSM_LR_	3	15.96%	0.79 (0.61–1.01)	0.061	0.0%	0.734	
PSM_RFA_ ≤ PSM_LR_	6	84.04%	0.93 (0.83–1.04)	0.200	53.3%	0.057	
**5-y OS**	11	100%	0.66 (0.52–0.85)	0.001	55.7%	0.012	
Publication year							**0.031**
Year (2003–2010)	6	31.52%	0.55 (0.43–0.75)	0.000	37.8%	0.154	
Year (2011–2018)	5	68.48%	0.90 (0.75–1.07)	0.217	0.0%	0.479	
Geographic location							0.183
Asian	5	55.71%	0.88 (0.72–1.06)	0.184	32.3%	0.206	
American	5	43.31%	0.67 (0.64–0.83)	0.000	55.4%	0.062	
European	1	0.98%	0.23 (0.05–0.98)	0.047	–	–	
Tumor size for RFA							0.631
≤ 3 cm	9	85.7%	0.77 (0.66–0.90)	0.001	61.1%	0.008	
>3 cm	2	14.3%	0.78 (0.53–1.15)	0.207	0.0%	0.550	
RFA methods							0.323^*^
Percutaneous	3	21.66%	0.51 (0.34‘0.75)	0.001	0.0%	0.536	
Intraoperative	5	78.34%	0.76 (0.62–0.93)	0.009	57.5%	0.052	
Adjuvant chemotherapy							0.296^*^
PAC_RFA_ > PAC_LR_	6	78.34%	0.84 (0.71–1.01)	0.059	62.3%	0.021	
PAC_RFA_ ≤ PAC_LR_	3	21.66%	0.53 (0.37–0.75)	0.000	0.0%	0.962	
Synchronous metastasis							0.039^*^
PSM_RFA_ > PSM_LR_	3	11.47%	0.38 (0.24–0.61)	0.000	0.0%	0.508	
PSM_RFA_ ≤ PSM_LR_	6	88.53%	0.84 (0.71–1.00)	0.050	35.3%	0.172	
**Complications**	8	100%	0.34 (0.23–0.51)	0.000	32.4%	0.170	

PAC, the percentage of patients received with adjuvant chemotherapy in RFA group or LR group; PSM, the percentage of patients with synchronous metastasis in RFA group or LR group; HR, hazard ratio; CI, confidence interval; Ph, P value of Q-test for heterogeneity test; Pr, P-value of meta regression analysis;

(*) *refer to the subgroup analysis and meta-regression analysis for patients reported relevant results; the influencing factors marked by the bold values in Pr column were regarded as the reason of heterogeneity by meta-regression analysis*.

### Complications

Eight of the included studies compared the complications between the RFA group and the LR group. The incidence of postoperative complication was significantly lower in the RFA group than that in the LR group (RR: 0.340, 95% CI: 0.230–0.510, *P* = 0.000, *I*^2^ = 32.4%, Ph <0.170) (Figure 2D, Table 2).

### Subgroup Analysis and Meta-Regression

**Figure 3:**
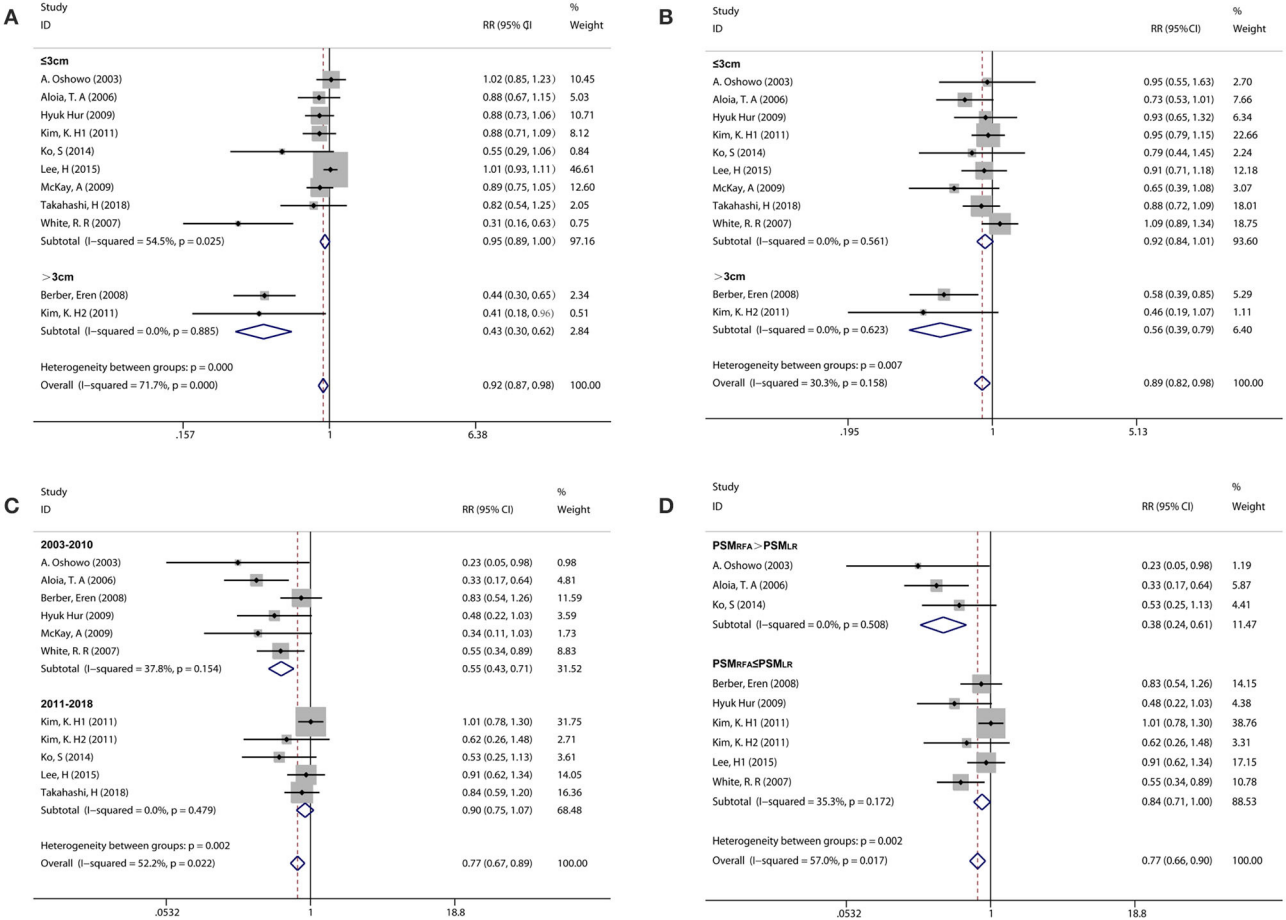
Subgroup analysis comparing the survival rate between RFA and LR groups. **(A)** Subgroup analysis for tumor size in the 1-year PFS. **(B)** Subgroup analysis for tumor size in the 3-year OS. **(C)** Subgroup analysis for publication year in the 5-year OS. **(D)** Subgroup analysis for the percentage of patients with synchronous metastasis in the 5-year OS.

Considering an existing difference among the studies and the differences among participants could contribute to overall heterogeneity among the included studies; the subgroup analysis was used to examine possible relationships between the study characteristics and 1-year PFS, 3-year OS, and 5-year OS. The subgroup analysis underlined several variables that could affect overall heterogeneity and influence the results of this meta-analysis. Namely, these were the publication years 2011–2018, geographic location (Asian), tumor size for RFA ( ≤ 3 cm), and adjuvant chemotherapy [the percentage of patients with adjuvant chemotherapy (PAC); PAC_RFA_ > PAC_LR_] in 1-year PFS; the geographic location (Asian), tumor size for RFA (≤ 3 cm), and adjuvant chemotherapy (PAC_RFA_ ≤ PAC_LR_) in 3-year OS; the publication years 2011–2018, geographic location (Asian), tumor size for RFA (>3 cm), adjuvant chemotherapy (PAC_RFA_ > PAC_LR_), and synchronous metastasis (the percentage of patients with synchronous metastasis, PSM; PSM_RFA_ > PSM_LR_) in 5-year OS (Figures 3A–D, Table 2). In addition, to further confirm the reason of heterogeneity, a meta-regression analysis was performed with predefined variables. The results of the meta-regression analysis also confirmed the obtained clarification of heterogeneity proposed by the subgroup analysis at several aspects, such as tumor size for RFA (Figures 3A,B, Table 2), publication year (Figure 3C, Table 2), and the percentage of patients with synchronous metastasis (Figure 3D, Table 2).

### Publication Bias

**Figure 4:**
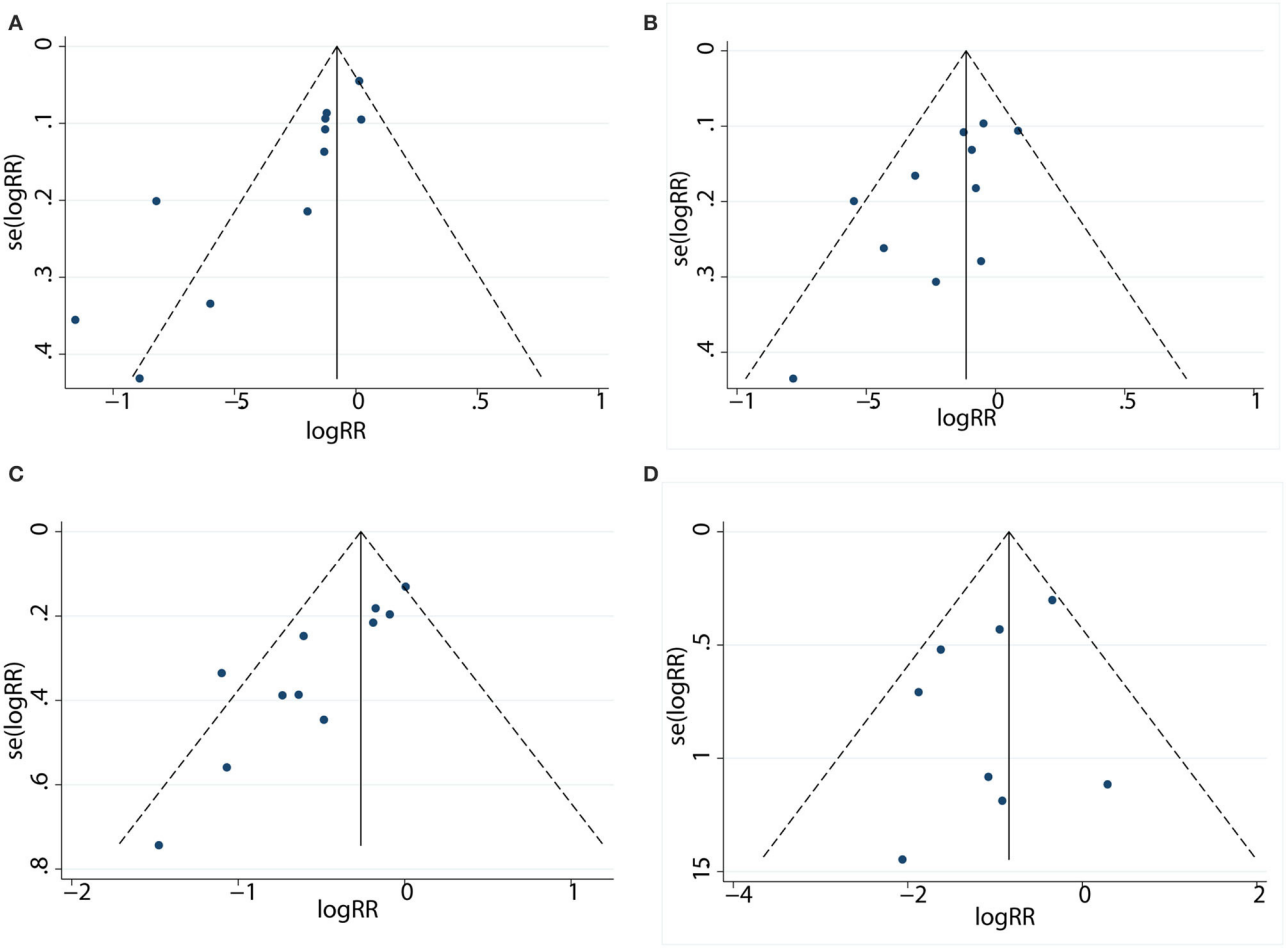
Funnel plot to detect publication bias between patients in the LR and RFA. **(A)** Funnel plot describing the comparative analysis of 1-year PFS rate. **(B)** Funnel plot describing the comparative analysis of 3-year OS rate. **(C)** Funnel plot describing the comparative analysis of 5-year OS rate. **(D)** Funnel plot describing the comparative analysis of complication rate.

The funnel plot did not show significant asymmetry by the Begg's test in 1-year PFS (Pr > |z| = 0.016) (Figure 4A), 3-year OS (Pr > |z| = 0.073) (Figure 4B), 5-year OS (Pr > |z| = 0.016) (Figure 4C), and complication rates (Pr > |z| = 1.000) (Figure 4D).

## Discussion

This meta-analysis examined published data and evidence obtained from relevant clinical trials to provide pooled estimates regarding the treatment efficacy between RFA and LR in CRLMs. In the present meta-analysis, the results found that patients with CRLMs who were treated by LR achieved better survival outcomes than those who were treated by RFA. However, RFA outperformed LR in terms of fewer perioperative complication rates.

In the meta-analysis, the inferior survival outcomes of RFA could be explained as follows. First, RFA patients in the included studies were not eligible for liver resection because of poor health status, prohibitive comorbidity, extrahepatic disease, multiple metastasis, inadequate liver remnant, etc. The poor basic condition may shorten the overall survival for patients with CRLMs. Second, there were several complex characteristics for tumors treated with RFA, such as close to the major vessel, larger lesion size, and difficult anatomical location. These characteristics increased the possibility of incomplete ablation for tumors, which would further accelerate the risk of tumor recurrence after RFA.

Because of the significant heterogeneity among the included studies, subgroup analysis and meta-regression were carried on. Considering the previously mentioned fact that incomplete ablation resulting from larger tumor size induced the expansion of tumor-initiating cells (16, 30), a subgroup analysis was performed for the influence of tumor size on RFA outcomes. The results showed that RFA patients with no more than a 3-cm tumor can achieve equivalent outcomes when compared with LR patients. In addition, the data from the meta-regression also displayed that the tumor size had an obvious influence on the significant heterogeneity in 1-year PFS and 3-year OS. According to the influence of tumor size on HCC outcomes, the reason for this may be explained by patients with smaller lesions achieving a higher ablation success rate, which can reduce the tumor recurrence attributed to hypoxia-driven acceleration of tumor growth occurring in the transition zone and the stimulated outgrowth of perilesional micrometastases (31, 32).

In contrast to the previous meta-analysis, this study also added the subgroup analysis about the publication year. Subgroup analysis showed that RFA patients in the publication years 2011–2018 had better survival than that in the publication years 2003–2010 and was identical when compared with LR patients in publication years 2011–2018. In addition, the meta-regression process in 5-year OS also further confirmed the subgroup analysis results. The results, to some extent, may be due to continuous improvement in the technical accuracy and performance of RFA in accordance with the learning curve for the treatment of CRLM, and the improvement process may be consistent with that in HCC treatment. In the past decades, RFA techniques have rapidly worked their way into clinical guidelines for the treatment of HCC, especially solitary small HCC. The international guidelines have shifted from surgical resection to minimally invasive percutaneous local ablation for small HCC (14, 33–35). Taken together, it will be possible that the therapeutic efficacy in the future will be better with the maturity of RFA technology in CRLM.

Meanwhile, to validate the effect of synchronous liver metastases on RFA therapeutic efficacy, this study also created an additional subgroup analysis and meta-regression. The results showed that synchronous liver metastases had a negative effect on the RFA therapeutic efficacy through subgroup analysis and meta-regression, which may be explained by cancer biology. According to the previous studies, the results showed that synchronous liver metastases have less favorable cancer biology and expected survival, and data from the corresponding registry showed that 5-year survival rates were shorter with synchronous than with metachronous CRLMs (36, 37). Yet, no biological marker has been identified that distinguishes synchronous metastases from metachronous metastases. Therefore, further research for biological markers is of significant importance for achieving better outcomes.

In addition, one of the results worth considering in the subgroup analysis was the finding that the Asian population can achieve better outcomes than the western population for RFA patients. Although, to the best of our knowledge, no exact evidence could be found to explain this, considering this observation, we propose the existence of a certain genetic variation among different ethnic groups. This was consistent with the fact that genome-wide polymorphism data have clearly established differences in allele frequency among continental regions (38). For the influence of gene mutation on CRLM, previous studies had demonstrated that KRAS mutation was associated with worse disease-free and overall survival following CRLM resection (39–41). However, previous reports showed that CRLM patients in Asian countries have similar KRAS mutation frequency when compared with those in western countries, and the morbidity and mortality of colorectal cancer in Asian countries were different from those in Western countries (42–44). Considering these facts, the result related to certain ethnic groups should only be used to generate a hypothesis that other genes, except the KRAS gene, may affect the outcomes for CRLM patients, which will be investigated in future research.

Of course, this meta-analysis has several limitations. First, all the included studies were retrospectively performed, which were susceptible to several biases. Second, significant heterogeneity was noticed, although the random effect model was used to compensate for part of the interstudy heterogeneity. In addition, the subgroup analysis in the adjuvant chemotherapy and RFA route should be interpreted with caution, which was mainly due to the failure of the meta-regression process to confirm the subgroup analysis results. In addition, the high-quality randomized controlled trails should be needed to resolve this problem and provide us with much more sound clinical evidences. Finally, publication bias remains to be a main concern; the Begg rank correlation for studies that involved comparative studies about therapeutic efficacy between RFA and LR suggested the presence of publication bias in 1-year PFS and 5-year OS. As we all know, articles with negative results were much more difficult to be published, and the majority of the included studies were from the surgery department; thus, the therapeutic efficacy of LR may be overvalued to some extent. In addition, although we tried to search for more relevant studies, the included number of studies may still be insufficient.

In conclusion, although LR was superior to RFA in the treatment of solitary CRLM in the meta-analysis, the subgroup analysis and meta-regression showed that the therapeutic efficacy of RFA was equivalent to that of LR in solitary CRLM, even when conditions were limited to tumors of ≤ 3 cm and fewer synchronous metastases in the publication year 2011–2018. Meanwhile, RFA provided lower rates of morbidities when compared with LR. In addition, further explanation should be interpreted through high-quality RCTs.

## Data Availability Statement

All datasets generated for this study are included in the article/ supplementary material.

## Author Contributions

The authors were responsible for the study design, data collection and analysis, preparation of the manuscript, and the final publishing decision. YK and YW designed the study, made a critical assessment, and had overall responsibility for this study. WH and JB conducted the literature review and data extraction, performed the statistical analysis, interpreted the statistical results, and wrote the manuscript. All authors read and approved the final manuscript.

## Conflict of Interest

The authors declare that the research was conducted in the absence of any commercial or financial relationships that could be construed as a potential conflict of interest.
